# Response to: Limbic system damage following SARS-CoV2 infection

**DOI:** 10.1093/braincomms/fcad342

**Published:** 2023-12-07

**Authors:** Philippe Voruz, Alexandre Cionca, Frédéric Assal, Julie A Péron

**Affiliations:** Clinical and Experimental Neuropsychology Laboratory, Faculty of Psychology, University of Geneva, Geneva 1205, Switzerland; Neurosurgery Department, Geneva University Hospitals, Geneva 1205, Switzerland; Clinical and Experimental Neuropsychology Laboratory, Faculty of Psychology, University of Geneva, Geneva 1205, Switzerland; Axon la, Radiology Department, Vaud University Hospital Center, Lausanne 1011, Switzerland; Neurology Department, Geneva University Hospitals, Geneva 1205, Switzerland; Faculty of Medicine, University of Geneva, Geneva 1205, Switzerland; Clinical and Experimental Neuropsychology Laboratory, Faculty of Psychology, University of Geneva, Geneva 1205, Switzerland; Neurology Department, Geneva University Hospitals, Geneva 1205, Switzerland

We express our gratitude to Taskiran-Sag and Yazgi for their letter^1^ concerning the recent article by Thomasson *et al*.^2^ in *Brain Communications*. Through this brief response, we aim to engage in a discussion on the thought-provoking points raised by the authors. The input from peers is crucial for both advancing our discipline and enhancing the management strategies for the post-COVID-19 scenario.

To begin, we share our colleagues’ viewpoint regarding the potential long-term neurological and neuropsychological effects of SARS-CoV-2 infection. While our article assessed the effects 6–9 months post-infection, it is crucial to note that the COVID-COG project (short and long-term neuropsychological impairment following COVID-19) is a broader initiative, of which Thomasson *et al*.'s study^2^ is a subset. This project aims to evaluate neuropsychological consequences up to 12–15 months post-infection. Consequently, we recently published the results of the second longitudinal patient visit, highlighting the persistence of cumulative neuropsychological deficits in the moderate and severe patient groups, in particular executive and memory deficits, along with the emergence of instrumental disorders such as language and perception in some patients.^3^ Moreover significant longitudinal relationships were observed between cumulated and self-reported depressive symptoms.^3^ In consideration of the aetiology of these neurocognitive disorders, and notably the hypothesis of the neurotropic effects of SARS-CoV-2, we appreciate this perspective and believe it is valuable to explore, not only direct impacts, but also potential indirect effects. There is merit in investigating pathways suggested in the literature, such as the influence on the nervous system through immune mechanisms, as also observed by our group.^4^ Given the design of the COVID-COG project, it may be challenging to provide a comprehensive answer to the specific question regarding neurotropism and other projects should explore this question further.

Secondly, and regarding the control group, we acknowledge this limitation and have endeavoured to address it within the article. The inclusion of a control group in the COVID-COG protocol posed challenges due to obvious ethical considerations. We were not authorized to conduct research on healthy individuals in Switzerland during this period; civilians and non-medical individuals were restricted from entering the Geneva University Hospital during this period. Moreover, and given the prevalence of the virus, it seems challenging to conceive a relevant post-pandemic control group, unfortunately. This is where the invaluable role of biobanks, such as those utilized by Douaud, Lee, Alfaro-Almagro, Arthofer, Wang, McCarthy, Lange, Andersson, Griffanti, Duff, Jbabdi, Taschler, Keating, Winkler, Collins, Matthews, Allen, Miller, Nichols and Smith^5^ becomes apparent, making this study a unique model in the scientific literature on post-COVID conditions. Unfortunately, these biobanks often lack a comprehensive neuropsychological evaluation such that of COVID-COG, especially concerning cognitive processes like emotional functions, or olfaction. Nevertheless, we were able to make behavioural comparisons with healthy individuals by analysing Geneva Emotion Recognition Test – Short version (GERT-S) results, juxtaposed with data from a cohort of healthy subjects at the onset of the pandemic.^6^ Standardized analyses indicated a higher prevalence of emotion recognition deficits in the moderate and severe groups. To compensate for the lack of control data, we also employed Monte Carlo simulations based on neuropsychological data. This approach allowed us to assess cumulative neuropsychological deficits in comparison with a simulated normative population.^7^ The results revealed significantly elevated cumulative neuropsychological deficits in the moderate and severe groups, although not in the mild group.^3^ It is important to note that while some individuals in the mild group exhibited deficits, these differences did not reach statistical significance at the group level.

Thirdly, the inquiries regarding structural effects on the limbic system are valid and align with existing literature. Notably, the voxel-based morphometric analyses did not reveal sufficiently significant differences among our patient groups. To clarify, the voxel-based morphometric analysis encompassed all brain regions based on our custom parcellation, which includes 100 cortical, 34 cerebellar, and 22 subcortical regions, with structures like the insula and hippocampus; Supplementary Table 4 presented only the most significant regions in the group comparison. This absence of effect has been corroborated in a recent article featuring a larger cohort.^8^ Acknowledging the potential influence of a control group on results, we also maintain that the severity of SARS-CoV-2 infection may not be the most reliable predictor of cognitive deficits. Consequently, we delved into patient phenotypes in *post hoc* studies, based on the current cohort.^4,9^ As the authors rightly point out, our findings suggest the potential existence of distinct patient phenotypes, either indicative of neurodegenerative processes (as supported by recent studies indicating an acceleration of the neurodegenerative cascade following SARS-CoV-2 infection),^10^ or symptoms akin to those observed in myalgic encephalomyelitis/chronic fatigue syndrome. Further exploration of these phenotypes, and their unique trajectories, necessitates long-term longitudinal studies. Fortunately, our group has secured the opportunity to extend the longitudinal evaluation of our cohort over 6 years and expand it, thanks to support from the Swiss National Research Fund (SNSF). This initiative, entitled TRAJECTORY, is set to begin in 2024.

Fourth, as highlighted by our colleagues, the enduring consequences of SARS-CoV-2 extend beyond the medical realm and manifest societal implications. This includes a potentially substantial mid- and long-term economic burden arising from the effects of cognitive impairment within the context of the post-COVID-19 condition.^11^

In conclusion, we extend our gratitude to the authors once more for their insightful letter, providing an opportunity for us to offer clarifications on our study and engage in discussions on a crucial theme that our disciplines are likely to confront in the future.

## Data Availability

Data sharing is not applicable to this article as no new data were created or analysed.
